# Acute high‐intensity muscle contraction moderates AChR gene expression independent of rapamycin‐sensitive mTORC1 pathway in rat skeletal muscle

**DOI:** 10.1113/EP091006

**Published:** 2024-11-05

**Authors:** Yuhei Makanae, Satoru Ato, Karina Kouzaki, Yuki Tamura, Koichi Nakazato

**Affiliations:** ^1^ Department of Physical Education National Defence Academy Yokosuka Japan; ^2^ Department of Life Science and Applied Chemistry Nagoya Institute of Technology Nagoya Japan; ^3^ Japan Society for the Promotion of Science Tokyo Japan; ^4^ Healty Food Science Research Group Cellular and Molecular Biotechnology Research Institute, Industrial Science and Technology (AIST) Tsukuba Japan; ^5^ Faculty of Health and Sports Sciences Toyo University Tokyo Japan; ^6^ Faculty of Medical Science Nippon Sport Science University Tokyo Japan; ^7^ Graduate School of Health and Sport Science Nippon Sport Science University Tokyo Japan

**Keywords:** acetylcholine receptor, mTORC1, muscle contraction

## Abstract

The relationship between mechanistic target of rapamycin complex 1 (mTORC1) activation after resistance exercise and acetylcholine receptor (AChR) subunit gene expression remains largely unknown. Therefore, we aimed to investigate the effect of electrical stimulation‐induced intense muscle contraction, which mimics acute resistance exercise, on the mRNA expression of AChR genes and the signalling pathways involved in neuromuscular junction (NMJ) maintenance, such as mTORC1 and muscle‐specific kinase (MuSK). The gastrocnemius muscle of male adult Sprague–Dawley rats was isometrically exercised. Upon completion of muscle contraction, the rats were euthanized in the early (after 0, 1, 3, 6 or 24 h) and late (after 48 or 72 h) recovery phases and the gastrocnemius muscles were removed. Non‐exercised control animals were euthanized in the basal state (control group). In the early recovery phase, *Agrn* gene expression increased whereas *LRP4* decreased without any change in the protein and gene expression of AChR gene subunits. In the late recovery phase, *Agrn*, *Musk*, *Chrnb1, Chrnd* and *Chrne* gene expression were altered and agrin and MuSK protein expression increased. Moreover, mTORC1 and protein kinase B/Akt‐histone deacetylase 4 (HDAC) were activated in the early phase but not in the late recovery phase. Furthermore, rapamycin, an inhibitor of mTORC1, did not disturb changes in AChR subunit gene expression after muscle contraction. However, rapamycin addition slightly increased AChR gene expression, while insulin did not impact it in rat L6 myotube. These results suggest that changes in the AChR subunits after muscle contraction are independent of the rapamycin‐sensitive mTORC1 pathway.

## Abstract (highlights)

- **What is the central question of this study?**
  Can muscle contraction‐induced activation of mechanistic target of rapamycin complex 1 (mTORC1) be linked to changes in acetylcholine receptor (AChR) gene expression and molecular signalling for maintaining neuromuscular junctions (NMJs)?
- **What is the main finding and its importance?**
  An acute high‐intensity muscle contraction dynamically changes AChR gene expression and muscle‐specific kinase (MuSK) signalling proteins at the late recovery phase of the contraction. Additionally, pharmacological inhibition of mTORC1 via rapamycin does not affect muscle contraction‐induced alterations in AChR genes. Our findings indicate that an acute high‐intensity muscle contraction induces changes in MuSK signalling and mTORC1‐independent changes in expression of AChR genes.

## 1 INTRODUCTION

The neuromuscular junction (NMJ) is the synapse between the motor nerve endings and myofibre that transmits nerve excitation to the myofibre, causing muscle contractions. Ageing‐associated NMJ disruption occurs before the loss of motor neuron cell bodies in the spinal cord (Chai et al., 2011). It has also been observed in amyotrophic lateral sclerosis (ALS) (Cappello & Francolini, 2017), myasthenia gravis (Howard, 2018) and muscular dystrophies (Rudolf et al., 2014). Therefore, NMJ maintenance is a crucial strategy against age‐related muscle weakness, and research into methods to maintain or improve NMJs is required.

Resistance training develops the nervous system and increases muscle mass, thereby improving muscle strength (Neto et al., 2022). In the early phase of muscle adaptation to resistance training, improvement of the nervous system mainly contributes to enhancing muscle strength. However, the mechanisms by which resistance training improves the nervous system in the early phase of adaptation remain unknown. A recent study indicated that an increase in motor neuron output to the muscle contributes to an increase in muscle strength after 4 weeks of resistance training (Del Vecchio et al., 2019). Additionally, recent studies in humans have shown that acute resistance exercise (Soendenbroe et al., 2020) and chronic resistance training alter the gene expression of acetylcholine receptor (AChR) subunits (Soendenbroe et al., 2020). Thus, changes in expression of AChR genes in response to resistance exercise may contribute to NMJ adaptation and increase muscle strength after resistance training because NMJs form AChR clusters in the myofibre.

Various signalling pathways are involved in NMJ maintenance such as AChR clustering via the muscle‐specific receptor tyrosine kinase (MuSK) pathway, a tyrosine receptor kinase signalling pathway that plays a crucial role in NMJ development (Herbst, 2020). Agrin binds to low‐density lipoprotein receptor‐related protein 4 (LRP4), which forms a complex with and activates MuSK. Activated MuSK induces AChR clustering. Additionally, Dok‐7, an adaptor protein that binds to MuSK, and rapsyn, a scaffolding protein for AChRs, are required for activating the MuSK signalling pathway and AChR clustering (Apel et al., 1997). In addition, MuSK protein expression was increased in a chronic training model that induces muscle hypertrophy in elderly rats (Bahreinipour et al., 2018). The mechanistic target of rapamycin complex 1 (mTORC1) pathway is also involved in NMJ maintenance (Baraldo et al., 2020; Castets et al., 2019; Ham et al., 2020). Muscle‐specific knockout of raptor, a scaffold protein of mTORC1, leads to NMJ fragmentation (Baraldo et al., 2020). Alternatively, genetically sustained activation of mTORC1 inhibits Akt activity through negative feedback and subsequently reduces the nuclear translocation of class II histone deacetylase 4 (HDAC), resulting in the reduction of AChR turnover after denervation (Castets et al., 2019). Additionally, overactivation of mTORC1 causes NMJ instability and impairs NMJ function (Ham et al., 2020). Thus, these studies indicate that proper mTORC1 activity is essential for the maintenance of NMJs.

Resistance exercise changes the activity of various signalling pathways, including mTORC1 (Burd et al., 2010; Ogasawara et al., 2016). A recent study demonstrated that Akt/mTOR/p70S6K signalling is involved in neureglin‐1‐induced upregulation of AChR subunit protein expression (Qiao et al., 2022). However, the relationship between the activation of mTORC1 after resistance exercise and AChR subunit gene expression remains largely unknown. Furthermore, although a previous study demonstrated that the inhibition of MuSK signalling through subcutaneous immunization by recombinant rat MuSK caused NMJ collapse and altered the regulation of the mTOR signalling pathway (Chauhan et al., 2013), suggesting a link between mTORC1 and MuSK signalling pathways, no study has directly investigated the relationship between mTORC1 and MuSK signalling pathways. The simultaneous identification of changes in AChR subunit gene expression and the signalling pathways involved in NMJ maintenance can contribute to the interpretation of the effect of resistance exercise on NMJ maintenance. Therefore, in this study, we investigated the effect of electrical stimulation‐induced muscle contraction, which mimics acute resistance exercise, on AChR subunit gene expression and signalling pathways involved in NMJ maintenance such as MuSK signalling.

## 2 METHODS

### 2.1 Ethical approval

Male Sprague–Dawley rats (10 weeks old) were obtained from CLEA Japan (Tokyo, Japan). All rats were housed for 1 week in an animal room with controlled temperature (22°C) and illumination (12/12 h light–dark cycle) before the study. The animals had access to commercial rat chow (CE7; CLEA Japan) and drinking water ad libitum. This study conformed to the ethical requirements outlined by the *Experimental Physiology* in accordance with guidelines for animal work (Grundy, 2015), and was approved by the Ethics Committee for Animal Experiments at Nippon Sports Science University (No. 020‐A04).

### 2.2 Electrical stimulation‐induced muscle contraction protocol

We used high‐intensity electrical stimulation‐induced muscle contraction, which mimics acute resistance exercise, as the experimental model. This model has been shown to induce mTORC1 activation and subsequent muscle hypertrophy (Adams et al., 2004; Ashida et al., 2018; Baar & Esser, 1999; O'Neil et al., 2009; Ogasawara et al., 2016, 2013). After an overnight fast (12 h), under isoflurane anaesthesia (approximately 2% concentration), the right lower leg of each rat was shaved and cleaned with alcohol wipes, and the animals were positioned with their right foot on a footplate (the ankle joint angle was 90°) in the prone posture on a fixing bed while maintaining body temperature. Anaesthetic depth was confirmed by the absence of nociceptive reflex. The triceps surae muscle was percutaneously stimulated with 10 mm × 5 mm electrodes (Vitrode V, Ag/AgCl; Nihon Kohden, Tokyo, Japan) connected to an electric stimulator and isolator (SS‐104J; Nihon Kohden) (Nakazato et al., 2010). The right gastrocnemius muscle was isometrically contracted (five sets of ten 3 s contractions, with a 7 s interval between contractions and 3 min rest intervals between sets), while the left gastrocnemius muscle served as an internal control. The voltage (∼30 V) and stimulation frequency (100 Hz) were adjusted to produce the maximal isometric tension (Makanae et al., 2019, 2015; Ogasawara et al., 2016).

In Experiment 1, rats were euthanized by exsanguination from the heart under deep isoflurane anaesthesia at 0, 1, 3, 6 or 24 h after completion of the muscle contraction, and the gastrocnemius muscles of both legs were removed (*n *= 5; each time point) to evaluate the response to acute muscle contraction in the early recovery phase. In Experiment 2, rats were euthanized by exsanguination from the heart under deep isoflurane anaesthesia at 48 or 72 h after completion of the muscle contraction, and the gastrocnemius muscles of both legs were removed (*n *= 5; each time point) to evaluate the response to acute muscle contraction in the late recovery phase. In both experiments, non‐exercised control animals were euthanized by exsanguination from the heart in a basal state (control group; *n *= 5) under deep isoflurane anaesthesia. In Experiment 3, the mTORC1 inhibitor rapamycin (1.5 mg/kg, 0.25 mg/mL in phosphate‐buffered saline (PBS) containing 5% dimethylsulfoxide (DMSO)) or placebo (PBS containing 5% DMSO) was intra‐peritoneally injected 1 h before and 48 h after exercise to evaluate the causal relationship between mTORC1 activation after muscle contraction and the expression of AChR genes. Rats were euthanized by exsanguination from the heart under deep isoflurane anaesthesia at 72 h after completion of the muscle contraction, and the gastrocnemius muscles of both legs were removed (*n *= 5; each time point). The tissues were rapidly frozen in liquid N_2_ and stored at −80°C until analysis.

### 2.3 Cell culture

Rat L6 cells were purchased from American Type Culture Collection (Manassas, VA, USA). Cells were seeded at a density of 2.0 × 10^4^ cells/cm^2^ and cultured for 3 days with growth medium (Dulbecco's modified Eagle's medium (DMEM), 10% fetal bovine serum, 1% penicillin/streptomycin) until subconfluent. After the 3‐day cultivation, the growth medium was replaced with a differentiation medium (DMEM, 2% horse serum, 1% penicillin/streptomycin), and cells were cultured for 9 days to form myotubes, by refreshing the medium every 48 h. The acute mTOR activation and/or inhibition experiment was performed on Day 9 post‐differentiation. The cells were serum‐deprived for 3 h followed by incubation with experimental regents. Four biological replicates were used for each condition.

### 2.4 Reagents

The treatment conditions of rapamycin and insulin were determined based on a previous study (O'Neil et al., 2009). Rapamycin was dissolved in DMSO at 10 mM as a stock solution. The stock solution was added to serum‐free DMEM to obtain a final concentration of 50 nM. Insulin was added to DMEM to prepare a 100 nM solution. The final DMSO concentration in the vehicle and insulin condition was adjusted to be similar to the rapamycin condition.

### 2.5 Western blotting

Western blot analysis was performed as previously reported (Makanae et al., 2019, 2015). Briefly, the frozen muscle samples were crushed with a liquid N_2_‐cooled pestle and mortar. Powdered muscle tissue and scraped cells were homogenized using a homogenizer in homogenization buffer containing 20 mM Tris‐HCl (pH 7.5), 1 mM Na_2_EDTA, 1% NP‐40, 2.5 mM sodium pyrophosphate, 1% sodium deoxycholate, 1 mM EGTA, 150 mM NaCl, 1 mM β‐glycerophosphate, 1 mM Na_3_VO_4_, 1 µg/mL leupeptin, and a protease and phosphatase inhibitor cocktail (Thermo Fisher Scientific, Waltham, MA, USA). The homogenates were centrifuged at 10,000 *g* for 10 min at 4°C. After the supernatant was removed, protein concentration was determined using a Protein Assay Rapid Kit (Fujifilm Wako Pure Chemical, Osaka, Japan). The samples were diluted in 3× sample buffer containing 15% v/v β‐mercaptoethanol, 6% w/v SDS, 187.5 mM Tris–HCl (pH 6.8), 30% v/v glycerol and 0.03% w/v bromophenol blue and boiled at 95°C for 5 min. Total proteins (10 µg) were separated through electrophoresis on 5–20% or 10–20% SDS–polyacrylamide gradient gels and electrophoretically transferred onto polyvinylidene difluoride (PVDF) membranes. After transfer, the membranes were washed in Tris‐buffered saline containing 0.1% Tween 20 (TBST) and blocked with PVDF blocking reagent (Amresco, Solon, OH, USA) for 5 min, with PVDF blocking regent for Can Get Signal (Toyobo, Osaka, Japan) for 1 h or with 5% skim milk in TBST for 1 h at room temperature. After blocking, membranes were washed and incubated with primary antibodies against agrin (cat. no. MAB5204; Merck, Darmstadt, Germany), LRP4 (cat. no. MAB5948; R&D Systems, Minneapolis, MN, USA), Dok‐7 (cat. no. sc390856; Santa Cruz Biotechnology, Dallas, TX, USA), phosphorylated‐MuSK (cat. no. ab192583; Abcam, Toronto, ON, Canada), MuSK (cat. no. sc134398; Santa Cruz Biotechnology), rapsyn (cat. no. ab156002; Abcam), phospho‐p70S6K (Thr389, cat. no. 9205), p70S6K (cat. no. 9202), phospho‐rpS6 (Ser240/244, cat. no. 2215), rpS6 (cat. no. 2317), phospho‐ULK1 (Ser317, cat. no. 12753; Thr757, cat. no.14202), ULK1 (cat. no. 8054), LC3 (cat. no. 2775), phospho‐Akt (Ser473, cat. no. 9271; Thr308, cat. no. 13038), Akt (cat. no. 9272), HDAC4 (cat. no. 2072), histone H3 (cat. no. 9715; Cell Signalling Technology, Danvers, MA, USA) and p62 (cat. no. PM045; Medical & Biological Laboratories, Aichi, Japan). Membranes were washed again in TBST and incubated with the appropriate secondary antibodies. Protein bands were detected using chemiluminescent reagents (Merck) and analysed by densitometry using a chemiluminescence detector (Fusion SOLO 6S EDGE; Vilber‐Lourmat, Marne‐la‐Vallée, France). The membranes were then stained with Ponceau S to verify equal loading in all the lanes and normalized by Coomassie brilliant blue staining if required. Band intensities were quantified using ImageJ software version 1.46 (National Institutes of Health, Bethesda, MD, USA).

### 2.6 Nuclear fractionation

Nuclear pellets were purified using a previously described method (Dimauro et al., 2012). Briefly, frozen and crushed muscle samples were placed in STM buffer comprising 250 mM sucrose, 50 mM Tris–HCl, pH 7.4, 5 mM MgCl_2_, protease and phosphatase inhibitor cocktails (Fujifilm Wako Pure Chemical) and thoroughly minced using scissors. All tissue samples were homogenized by 40 strokes using a Potter glass homogenizer. The tissue homogenates were centrifuged at 500 *g* for 15 min at 4°C, and the pellets were collected and resuspended in STM buffer and passed through a 40 mm cell strainer (Argos Technologies Inc., Vernon Hills, IL, USA). The lysates were centrifuged at 500 *g* for 15 min at 4°C, the pellets were collected and re‐suspended in STM buffer containing 10% Triton X‐100 and homogenized by 20 strokes using a Potter glass homogenizer. The lysates were centrifuged at 500 *g* for 15 min at 4°C, and the pellets were collected and washed once with STM buffer containing 10% Triton X‐100. Subsequently, the pellets were re‐suspended in nuclear extraction buffer, comprising 20 mM HEPES (Dojindo Molecular Technologies, Rockville, MD, USA), 1.5 mM MgCl_2_, 0.5 M NaCl (Fujifilm Wako Pure Chemical), 0.2 mM EDTA, 20% glycerol (Fujifilm Wako Pure Chemical) and 1% Triton X‐100, pH 7.9 (Fujifilm Wako Pure Chemical), and vortexed. The lysates were sonicated at a high setting for 30 s with 15 s pauses whilst being kept on ice‐cold water throughout and centrifuged at 9000 *g* for 30 min at 4°C. The resulting supernatants were collected and used as the final nuclear fractions. The protein concentrations of the nuclear fractions were determined, and the lysates were analysed through a western blot.

### 2.7 Real‐time reverse transcription polymerase chain reaction

Total RNA was extracted from the powdered muscle sample and cultured cells using TRIzol reagent (Thermo Fisher Scientific) according to the manufacturer's instructions. Total RNA concentrations were measured using a NanoDrop One spectrophotometer (Thermo Fisher Scientific), and 500 ng of total RNA was reverse‐transcribed into cDNA using ReverTra Ace RT Master Mix (Toyobo). The cDNA product was mixed with qPCR reagent (Thunderbird SYBR qPCR Mix, Toyobo) and primers and analysed using a thermal cycler (CFX96 Touch, Bio‐Rad Laboratories, Hercules, CA, USA) with an optical reaction module. Primer sequences used in this study are listed in Table 1. Gene expression was quantified using the calibration curve method. The housekeeping genes β2‐microgloblin (*B2M*) and hypoxanthine phosphoribosyltransferase 1 (*HPRT1*) were used as internal controls.

**TABLE 1:** Primer sequences for RT‐PCR.

Gene	Forword primer	Reverse primer
*Agrn*	CCTCAACTTGGACACGAAGCT	AGGCCGATGCCCACAGA
*Lrp4*	GATGAACGTAACTGCACCACC	TTCCAACACAGGAACTGGTCA
*Musk*	CCACTGTCAGTATAGCAGAATGGA	TTCACCAGGACGGCATCAC
*Dok7*	GTCTCCGCAGTGGAAGAGTAG	AGAAGAAGACTCCAGCCCAGTA
*Chrna*	TCCCTTCGATGAGCAGAACT	AGCCGTCATAGGTCCAAGTG
*Chrnb*	CCATGGTGTTTAGTTCCTCTT	CCTTCCCTCCTCTTC
*Chrnd*	TGTGGAGAGAAGACCTCG	AGCCTCTTGGAGATAAGCAAC
*Chrne*	CCGAGGTCTTCTCTCCACAG	ACCACCAAGACGTCACCTTC
*Rapsn*	TACGCCCAGGTCAAGGAGT	TCCGGTGGATATCGGCAAAG
*B2M*	GCTTGCCATTCAGAAAACTCC	AAGTTGGGCTTCCCATTCTC
*HPRT1*	CTCATGGACTGATTATGGACAGGAC	GCAGGTCAGCAAAGAACTTATAGCC

### 2.8 Statistical analyses

One‐way ANOVA was used to evaluate changes in protein, phosphorylated protein and mRNA expression in Experiment 1, 2 and the in vitro experiment. A two‐way ANOVA (rapamycin × contraction) was used to evaluate changes in protein and mRNA expression in Experiment 3. *Post hoc* analyses were performed using Student's *t*‐test, with Benjamini and Hochberg's false discovery rate correction for multiple comparisons when significant changes were found. All values are expressed as means with individual values in bar and dot plots. The level of significance was set at *P* < 0.05.

## 3 RESULTS

### 3.1 Experiment 1

#### 3.1.1 mRNA expression

**FIGURE 1:**
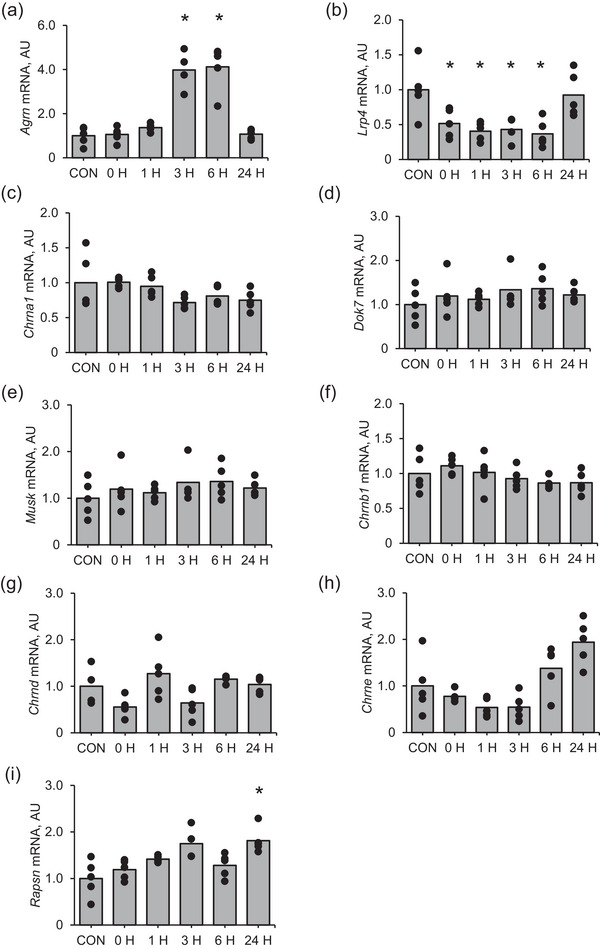
mRNA expression of NMJ‐related factors in the early recovery phase from an acute bout of muscle contraction. (a) *Agrn*, (b) *Lrp4*, (c) *Musk*, (d) *Dok7*, (e) *Chrna1*, (f) *Chrnb1*, (g) *Chrnd*, (h) *Rapsn* and (i) *Chrne*. Mean values are shown with individual values in bar and dot plots. *P < 0.05 versus CON. AU, arbitrary units; CON, control.

*Agrn* mRNA expression was significantly increased at 3 and 6 h after muscle contraction (*P* = 0.0003 and *P* = 0.0002, respectively) and returned to the control level at 24 h (*P* = 0.733) after muscle contraction compared with that in the control (Figure 1a). *Lrp4* mRNA expression was significantly lower at 0, 1, 3 and 6 h after muscle contraction (*P* = 0.036, *P* = 0.011, *P* = 0.003 and *P* = 0.011, respectively) than that in the control and returned to the control level at 24 h (*P* = 0.734) after muscle contraction (Figure 1b). *Rapsn* mRNA expression was significantly higher at 24 h after muscle contraction than that in the control (Figure 1i; *P* = 0.005). However, the changes in mRNA expression of *MuSK* (Figure 1c), *Dok7* (Figure 1d), *AChR* (*Chrn*)*a1* (Figure 1e), *Chrnb1* (Figure 1f), *Chrnd* (Figure 1g) and *Chrne* (Figure 1h) in response to muscle contraction were not significant.

#### 3.1.2 Protein expression

**FIGURE 2:**
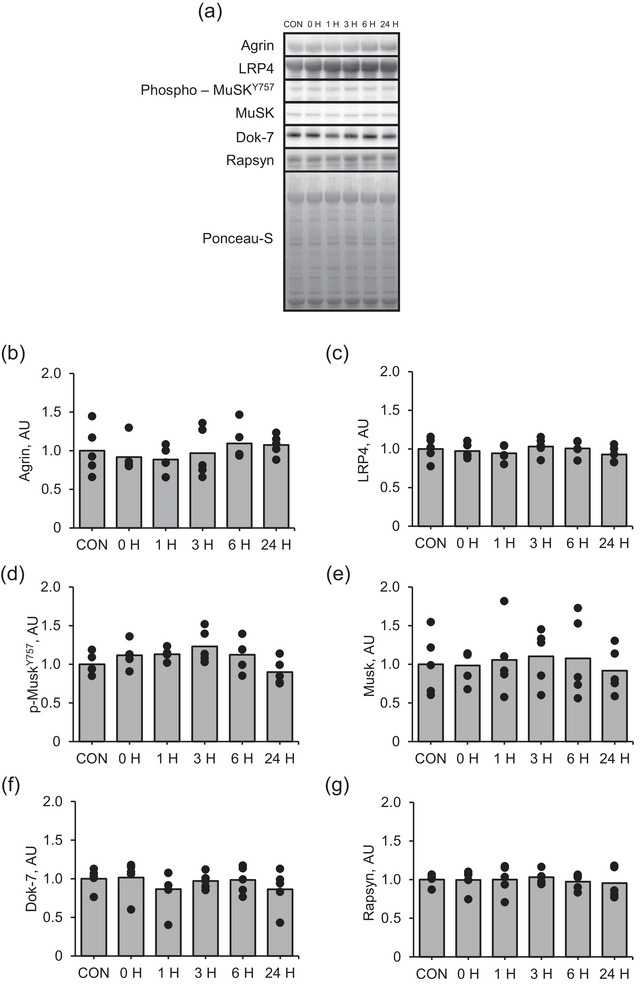
Protein expression of NMJ‐related factors in the early recovery phase from an acute bout of muscle contraction. (a) Representative images, (b) agrin, (c) Lrp4, (d) phosphorylated MuSK at Tyr755, (e) MuSK, (f) Dok‐7 and (g) rapsyn. Mean values are shown with individual values in bar and dot plots. *P < 0.05 versus CON. AU, arbitrary units; CON, control.

The changes in the expression of the measured NMJ‐related proteins, including agrin, LRP4, Dok‐7, phosphorylated‐MuSK, MuSK and rapsyn, in response to muscle contraction were not significant (Figure 2b–g).

**FIGURE 3:**
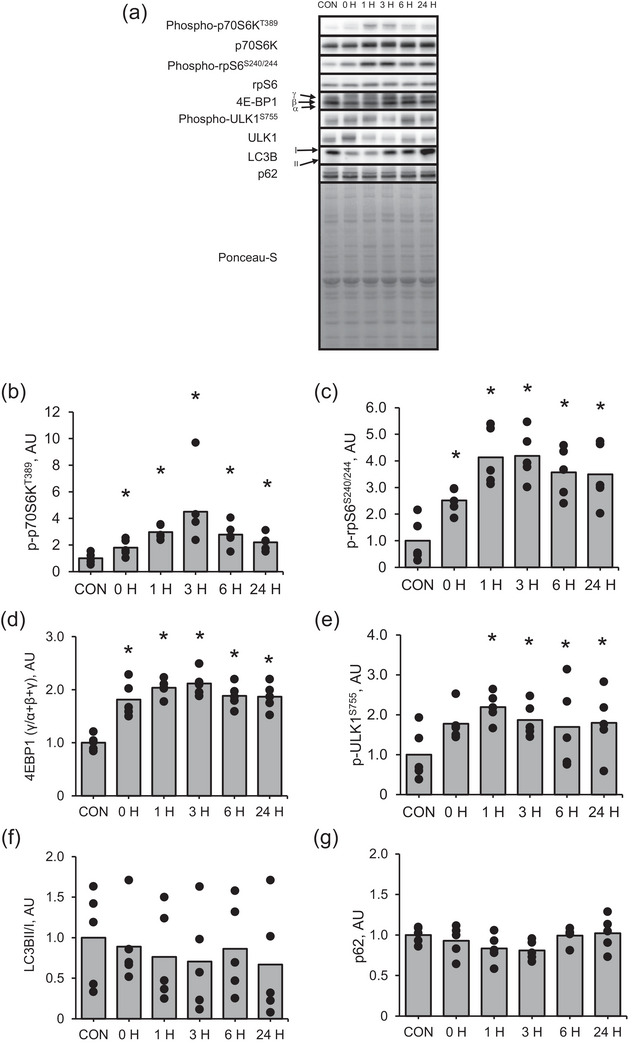
Protein expression of mTORC1 signalling pathway in the early recovery phase from an acute bout of muscle contraction. (a) Representative images, (b) phosphorylation of p70S6K at Thr389, (c) phosphorylation of rpS6 at Ser240/244, (d) 4EBP1 (γ/α + β + γ), (e) phosphorylation of ULK at Ser755, (f) LC3BI/II and (g) p62. Data of protein phosphorylation are presented as ratios relative to total protein. Mean values are shown with individual values in bar and dot plots. *P < 0.05 versus CON. AU, arbitrary units; CON, control.

We measured the expression of phosphorylated p70S6K and rpS6 and the expression of 4E‐BP1 in gamma (γ) form, which indicates the dissociation of 4E‐BP1 from eIF4E, to investigate mTORC1 activity. When 4E‐BP1 is bound to eIF4E, translation at ribosomes is limited; however, the inactivation of 4E‐BP1 leads to its disassociation from eIF4E and enhances translation at ribosomes. We found that the phosphorylation of p70S6K (Figure 3b) and rpS6 (Figure 3c) was significantly higher immediately (*P* = 0.046 and *P* = 0.008, respectively) after muscle contraction than that in the control and remained high at 24 h (*P* = 0.007 and *P* = 0.011, respectively) after muscle contraction. The expression of 4E‐BP1 in γ‐form was significantly higher immediately (*P* < 0.0001) after muscle contraction than that in the control and remained high at 24 h after muscle contraction (Figure 3d, P = 0.0002). We also measured the expression of phosphorylated ULK1 at Ser757, a downstream signal of mTORC1, and the LC3BII to I ratio and p62 expression, which are autophagy markers, to clarify the effect of muscle contraction on the relation between autophagy and NMJ‐related factors. The expression of phosphorylated ULK1 was significantly higher at 1 h after muscle contraction than in the control (Figure 3e, P = 0.007), and remained high at 24 h (*P* = 0.0002), and total ULK1 expression was significantly higher at immediately after muscle contraction than in the control (*P* = 0.004). The changes in LC3BII to I ratio (Figure 3f) or p62 protein expression (Figure 3g) in response to muscle contraction were not significant.

**FIGURE 4:**
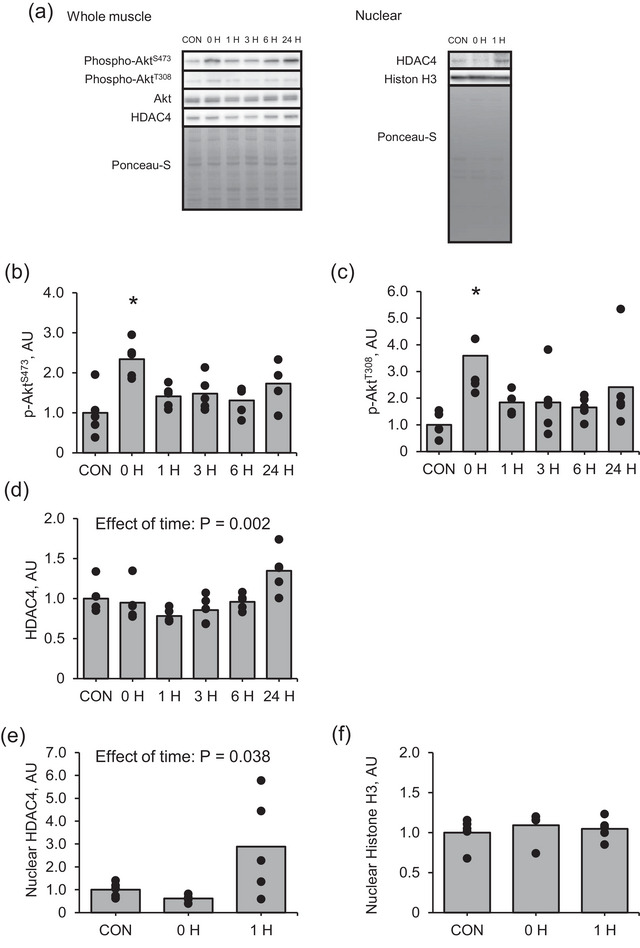
Protein expression of Akt–HDAC4 signalling pathway and intra‐nuclear HDAC4 in the early recovery phase from an acute bout of muscle contraction. (a) Representative images, (b) phosphorylation of Akt at Ser473, (c) phosphorylation of Akt at Thr308, (d) HDAC4, (e) intra‐nuclear HDAC4 and (f) intra‐nuclear histone H3. Data regarding protein phosphorylation are presented as ratios relative to total protein. Mean values are shown with individual values in bar and dot plots. *P < 0.05 versus CON. AU, arbitrary units; CON, control.

The phosphorylation of Akt at Ser473 was significantly higher immediately after muscle contraction than that in the control (Figure 4b, P = 0.017). Moreover, the phosphorylation of Akt at Thr308 was significantly higher immediately and 1 h after muscle contraction than in the control (Figure 4c, P = 0.007 and *P* = 0.002, respectively). Although the one‐way ANOVA revealed that muscle contraction influenced the intra‐muscular (Figure 4d, P = 0.002) and nuclear (Figure 4e, P = 0.033) HDAC4 protein expression, the difference in nuclear HDAC4 expression among groups was not significant.

### 3.2 Experiment 2

In Experiment 1, we observed mTORC1 activation and changes in NMJ stability‐related factors but did not observe changes in *Chrn* subunits. Previous human studies have demonstrated that the gene expression of *Chrn* subunits changes a few days after resistance exercise (Soendenbroe et al., 2020). Therefore, we investigated the effect of muscle contraction on NMJ‐related factors in the late recovery phase after muscle contraction, when mTORC1 activity returns to the basal condition.

#### 3.2.1 mRNA expression

**FIGURE 5:**
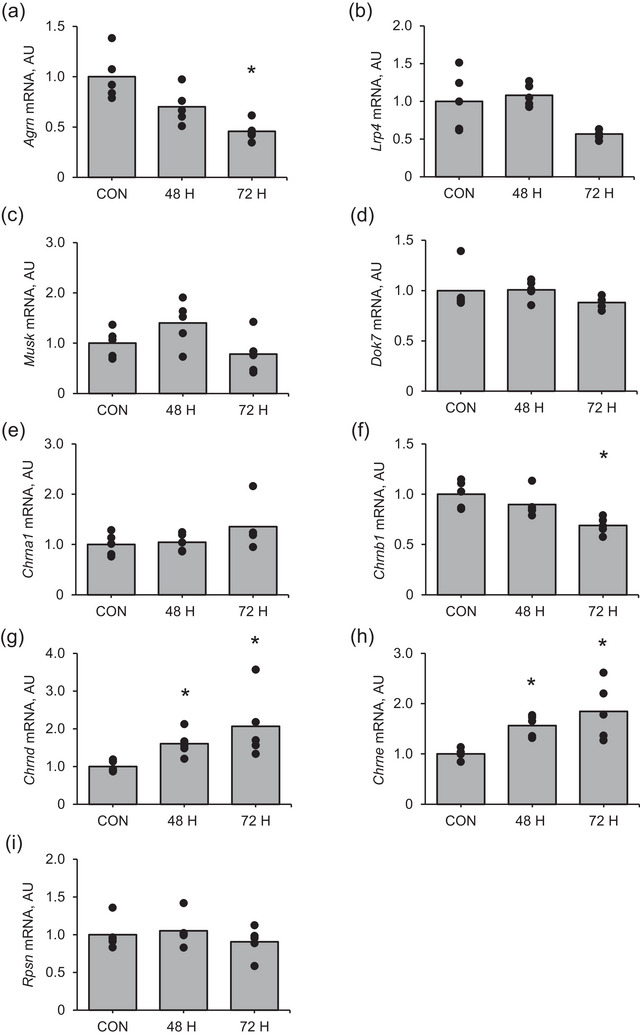
mRNA expression of NMJ‐related factors in the late recovery phase from an acute bout of muscle contraction. (a) *Agrn*, (b) *Lrp4*, (c) *Musk*, (d) *Dok7*, (e) *Chrna1*, (f) *Chrnb1*, (g) *Chrnd*, (h) *Rapsn* and (i) *Chrne*. Mean values are shown with individual values in bar and dot plots. *P < 0.05 versus CON. AU, arbitrary units; CON, control.

Compared with the control, *Agrn* mRNA expression significantly decreased at 72 h after muscle contraction (Figure 5a; *P* = 0.002). *Chrnb1* expression was significantly lower at 72 h after muscle contraction than in the control (Figure 5f; *P* = 0.002). In contrast, *Chrnd* (Figure 5g) and *Chrne* (Figure 5h) expression was significantly higher at 48 (*P* = 0.0062 and *P* = 0.0007, respectively) and 72 h (*P* = 0.03 and *P* = 0.011, respectively) after muscle contraction than in the control. However, the changes in *LRP4*, *Dok7*, *MuSK*, *Chrna1* or *Rapsn* mRNA expression in response to muscle contraction were not significant.

#### 3.2.2 Protein expression

**FIGURE 6:**
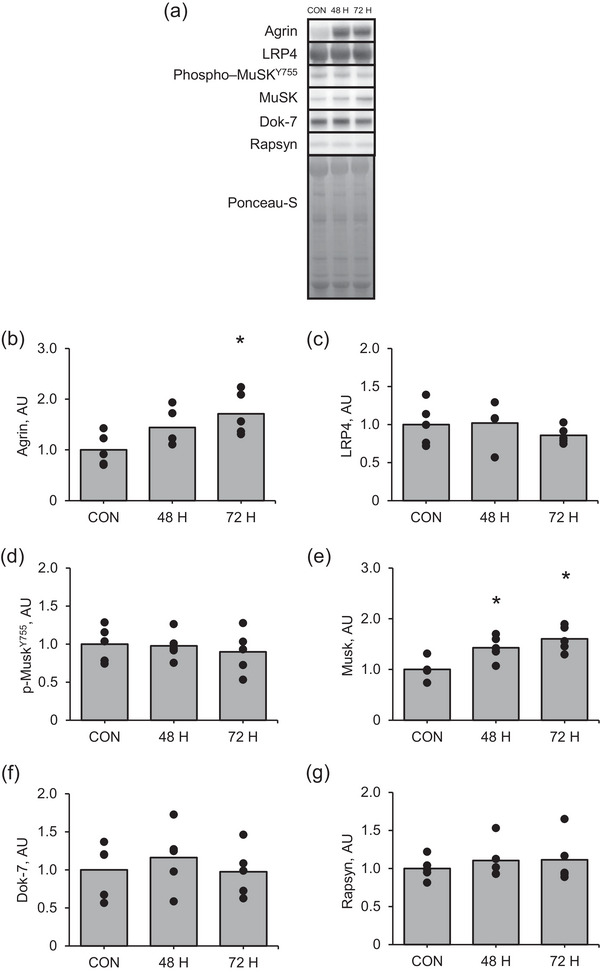
Protein expression of NMJ‐related factors in the late recovery phase from an acute bout of muscle contraction. (a) Representative images, (b) agrin, (c) Lrp4, (d) phosphorylated MuSK at Tyr755, (e) MuSK, (f) Dok‐7 and (g) rapsyn. Mean values are shown with individual values in bar and dot plots. *P < 0.05 versus CON. AU, arbitrary units; CON, control.

**FIGURE 7:**
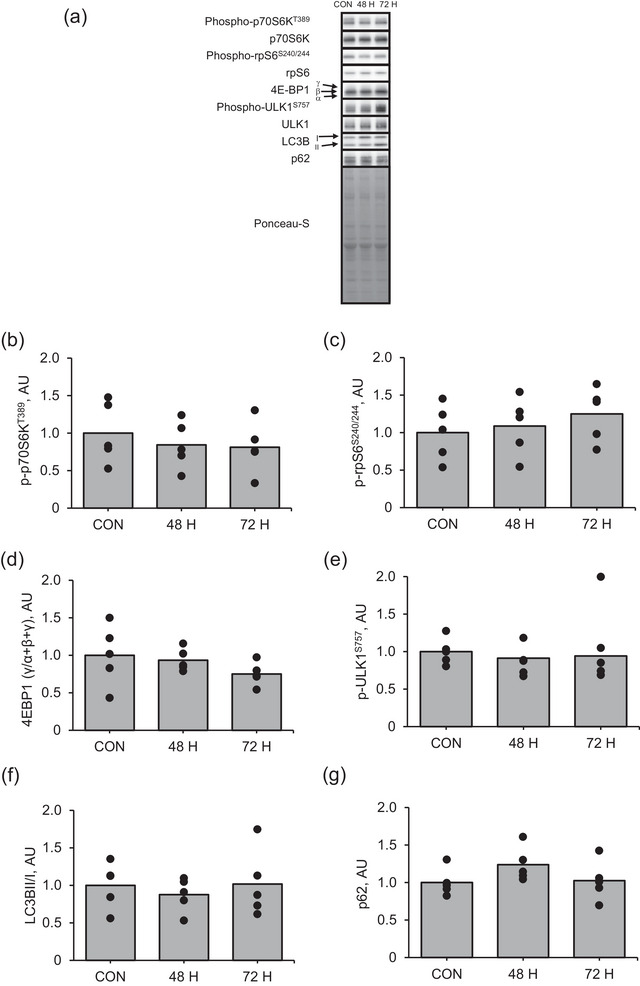
Protein expression of mTORC1 signalling pathway in the late recovery phase from an acute bout of muscle contraction. (a) Representative images, (b) phosphorylation of p70S6K at Thr389, (c) phosphorylation of rpS6 at Ser240/244, (d) 4EBP1 (γ/α + β + γ), (e) phosphorylation of ULK at Ser755, (f) LC3BI/II and (g) p62. Data regarding protein phosphorylation are presented as ratios relative to total protein. Mean values are shown with individual values in bar and dot plots. *P < 0.05 versus CON. AU, arbitrary units; CON, control.

Agrin expression was significantly higher at 72 h after muscle contraction than in the control (Figure 6b; *P* = 0.002). Additionally, MuSK expression was significantly higher at 48 and 72 h after muscle contraction than in the control (Figure 6e; *P* = 0.017 and *P* = 0.003, respectively). However, changes in LRP4 (Figure 6c), phosphorylated MuSK (Figure 6d), Dok‐7 (Figure 6f) or rapsyn (Figure 6g) protein expression in response to muscle contraction were not significant. Moreover, the changes in the expression of proteins related to mTORC1 activity, such as p70S6K, rpS6 and 4E‐BP1, and autophagy, such as ULK1, LC3BII/I and p62, at 48 and 72 h after muscle contraction were not significant (Figure 7b–g).

**FIGURE 8:**
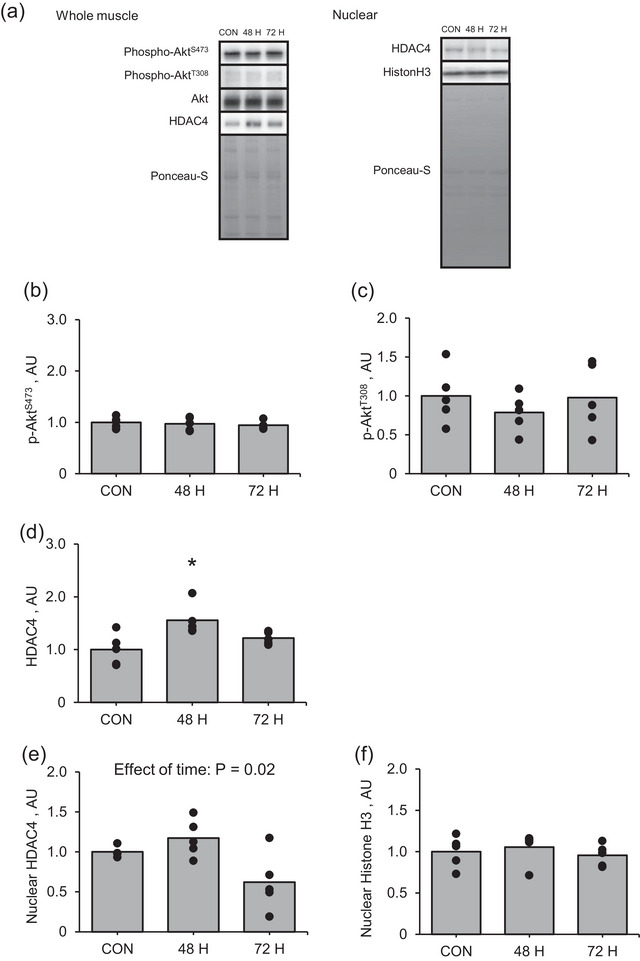
Protein expression of Akt–HDAC4 signalling pathway and intra‐nuclear HDAC4 in the late recovery phase from an acute bout of muscle contraction. (a) Representative images, (b) phosphorylation of Akt at Ser473, (c) phosphorylation of Akt at Thr308, (d) HDAC4, (e) intra‐nuclear HDAC4 and (f) intra‐nuclear histone H3. Data regarding protein phosphorylation are presented as ratios relative to total protein. Mean values are shown with individual values in bar and dot plots. *P < 0.05 versus CON. AU, arbitrary units; CON, control group.

Additionally, the changes in phosphorylation of Akt at both Ser473 and Thr308 at 48 and 72 h after muscle contraction were not significant (Figure 8b,c). In contrast, the intra‐muscular HDAC4 expression was significantly higher at 48 h after muscle contraction than in the control (Figure 8d; *P* = 0.009). However, the difference in nuclear HDAC4 expression after muscle contraction among groups was not significant even though the one‐way ANOVA revealed that muscle contraction influenced intra‐nuclear HDAC4 expression (Figure 8e, P = 0.02).

### 3.3 Experiment 3

**FIGURE 9:**
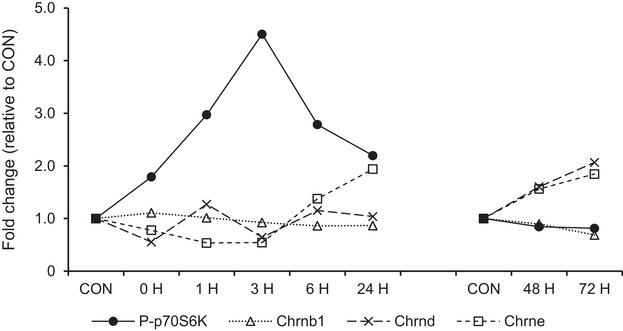
Time course of mTORC1 activation (p70S6K phosphorylation) and AChR gene expression and MuSK signalling. All data are presented as relative to control.

The results of Experiments 1 and 2 indicated that mTORC1 activation after muscle contraction did not contribute to changes in AChR subunit gene expression (Figure 9). Therefore, we investigated the effect of muscle contraction on AChR subunits under mTORC1 inhibition using rapamycin, an mTORC1 inhibiter. Furthermore, considering that rapamycin works on the whole body, we investigated whether mTORC1 affects expression of AChR genes in muscle cell culture using rapamycin and insulin, a stimulator of mTORC1.

#### 3.3.1 In vivo experiment

##### mRNA expression

**FIGURE 10:**
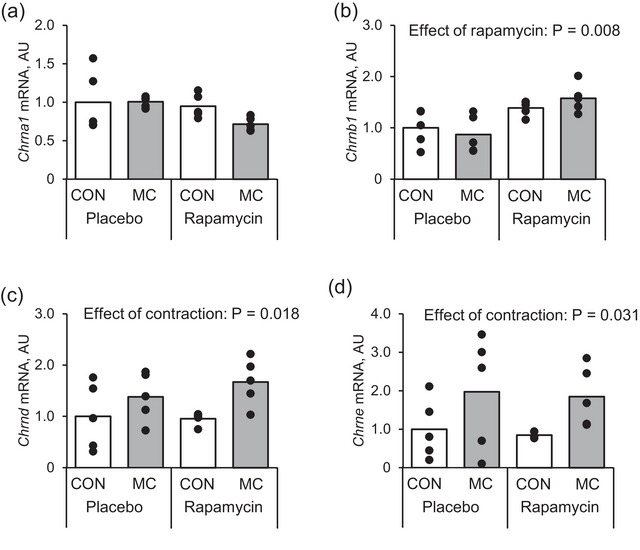
mRNA expression of AChR at 72 h after an acute bout of muscle contraction with rapamycin administration. (a) *Chrna1*, (b) *Chrnb1*, (c) *Chrnd* and (d) *Chrne*. Mean values are shown with individual values in bar and dot plots. AU, arbitrary units; CON, control leg; MC, muscle contraction.

The administration of rapamycin significantly increased in *Chrnb1* expression at 72 h after muscle contraction in the rats (Figure 10b; *P* = 0.0008). Although no significant effect of rapamycin on *Chrnd* (Figure 10c; *P* = 0.569) and *Chrne* (Figure 10d; *P* = 0.741) expression was found, muscle contraction significantly increased *Chrnd* (Figure 10c; *P* = 0.018) and *Chrne* (Figure 10d; *P* = 0.031) expression. Neither rapamycin (*P* = 0.966) nor muscle contraction (*P* = 0.054) changed the mRNA expression of *Chrna1* (Figure 10a). No significant interaction was observed between rapamycin and muscle contraction in all measured mRNA.

##### Protein expression

**FIGURE 11:**
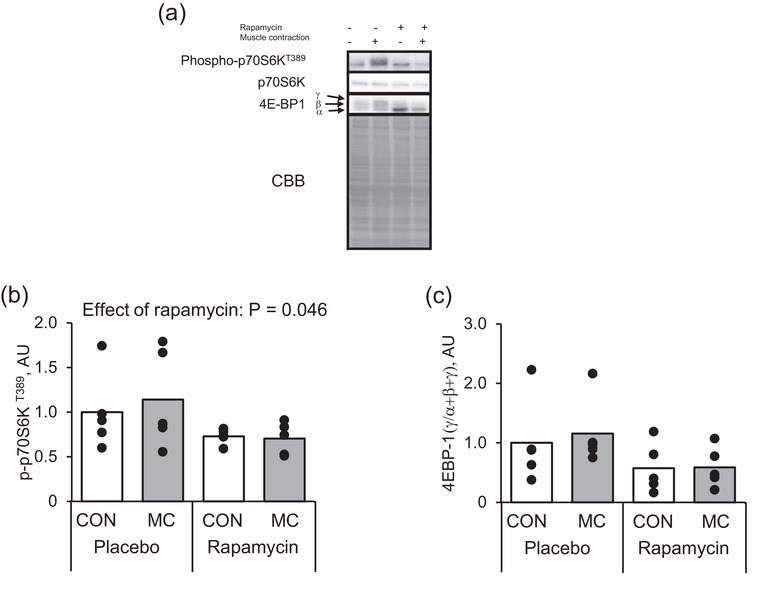
Protein expression of mTORC1 signalling pathway at 72 h after an acute bout of muscle contraction with rapamycin administration. (a) Representative images, (b) *Chrnb1*, (c) *Chrnd* and (d) *Chrne*. Mean values are shown with individual values in bar and dot plots. AU, arbitrary units; CON, control leg; MC, muscle contraction.

Muscle contraction did not affect mTORC1 activity indicators such as p70S6K (Figure 11b; *P* = 0.723) and 4E‐BP1 (Figure 11c; *P* = 0.725) at 72 h after muscle contraction. Additionally, rapamycin decreased phosphorylation of p70S6K Thr389 (Figure 11b; *P* = 0.046) but did not change 4E‐BP1 (Figure 11c; *P* = 0.052), corresponding with a previous study that demonstrated the effect of rapamycin on mTORC1 activity after muscle contraction (Ogasawara et al., 2016). No significant interaction was observed between rapamycin and muscle contraction in all measured proteins.

#### 3.3.2 In vitro experiment

**FIGURE 12:**
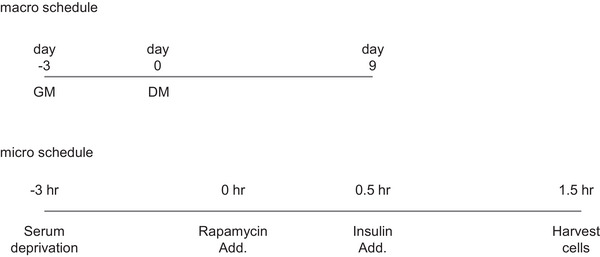
Schematic representation of the experimental protocol in L6 myotube. DM, differentiation medium; GM, growth medium.

**FIGURE 13:**
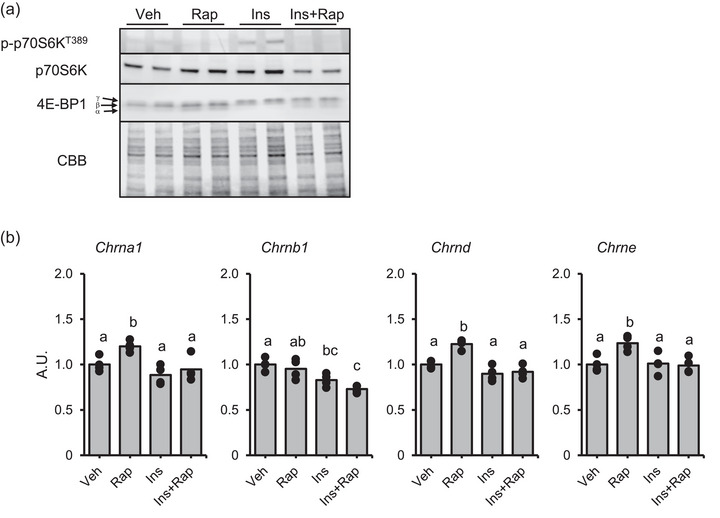
The effects of insulin and rapamycin on (a) mTORC1 signalling pathway and (b) AChR gene expression in L6 myotube. Values are shown as the mean with individual values in bar and dot plots. Statistically significant differences (*P* < 0.05) are represented by different letters. AU, arbitrary units; Ins, insulin; Rap, rapamycin; Veh, vehicle.

We then evaluated the mechanical relationship between mTORC1 activity and expression of AChR genes In vitro, although this was previously tested in several studies (Altiok et al., 1997; Si et al., 1996). The rat L6 myotubes were treated with rapamycin 30 min prior to insulin addition. Thirty minutes after the insulin addition, cells were harvested and analysed (Figure 12). Phosphorylation of p70S6K Thr389 by insulin treatment was observed while the combination with rapamycin successfully inhibited it. 4E‐BP1 hyperphosphorylation (upshift of the band) was confirmed by insulin treatment (Figure 13a). The rapamycin combination with insulin partially blocked 4E‐BP1 phosphorylation (i.e., visualized the α form of 4E‐BP1, Figure 13a). These results suggest that the rapamycin‐sensitive function of mTORC1 was sufficiently blocked. Expression of AChR genes was slight but significantly increased with rapamycin addition, while the insulin did not impact gene expression levels (Figure 13b).

## 4 DISCUSSION

In this study, we investigated time‐dependent changes in expression of AChR genes, MuSK signalling‐related factors and mTORC1 signalling in response to acute high‐intensity contractions in the skeletal muscle of rats. In the early recovery phase after muscle contraction, the gene expression of *Agrn* increased while that of *Lrp4* transiently decreased immediately and at 24 h after muscle contraction; however, the protein and gene expression of AChR subunits remained unchanged. Additionally, we found that muscle contraction had a greater effect on the factors related to NMJ maintenance and expression of AChR genes in the late recovery phase than in the early recovery phase. The expression of *Agrn*, *Musk*, *Chrnb1*, *Chrnd* and *Chrne* genes changed at 48 and 72 h after muscle contraction. Moreover, agrin and MuSK protein expression increased at 72 h after muscle contraction. To the best of our knowledge, this study is the first to demonstrate that intense muscle contraction that mimics acute resistance exercise can increase the mRNA and protein expression of both agrin and MuSK, which are required for NMJ maintenance. However, the timing of mTORC1 activation did not coincide with that of the changes in the gene expression of AChR subunits.

The nuclear translocation of HDAC4, through Akt activation, plays an essential role in the recovery of NMJs after denervation (Castets et al., 2019). In the present study, muscle contraction increased Akt phosphorylation and changed intra‐nuclear HDAC4 expression in the early recovery phase. We performed the experiment using rapamycin, an inhibitor of mTORC1, to determine whether the activation of mTORC1 involves the changes in AChR genes after muscle contraction. Rapamycin administration suppressed mTORC1 activity but did not inhibit an increase in expression of AChR genes after muscle contraction. Furthermore, our In vitro experiment supported the results observed in the in vivo rapamycin administration experiment and suggested that activation of rapamycin‐sensitive mTORC1 is not required for the increase in gene expression of the AChR subunits induced by muscle contraction.

### 4.1 Muscle contraction changed the expression of NMJ‐related factors

The AChR in the adult organism consists of four subunits, a, b, d and e (*Chrna*, *Chrnb*, *Chrnd* and *Chrne*). Our results showed that muscle contraction changed the mRNA expression of AChR genes in the late recovery phase. The expression of *Chrnd* and *Chrne* genes increased at 48 h after muscle contraction and remained elevated for 72 h after muscle contraction. In contrast, *Chrnb1* expression decreased at 72 h after muscle contraction. This is consistent with a previous study which demonstrated that the AChR gene response to exercise varied depending on the AChR subunits in young women (Soendenbroe et al., 2020). During early *Xenopus* development, myogenic regulatory factors (MRFs) such as MyoD, Myf‐5 and MRF4, which are exclusively expressed in adult myofibres, specifically regulate the transcriptional activation of genes encoding different subunits of AChR genes (Charbonnier et al., 2003). In addition, the forced expression of myogenin, an MRF that is expressed in mature myofibres, resulted in an increase in AChR gene expression in vivo (Gundersen et al., 1995). Therefore, MRFs might be involved in exercise‐induced AChR gene dynamics and should be further investigated. Although it is unclear whether the difference in the mRNA response of the AChR subunits to exercise has functional significance, a previous study has indicated that the increase in the AChR subunit mRNA plays a regulatory role in intra‐muscular AChR expression (Evans et al., 1987). Even though we did not measure the morphological changes in the NMJ in the present study, an acute bout of muscle contraction may induce NMJ remodelling. Therefore, further histochemical studies are required to confirm this hypothesis and to clarify the effects of chronic resistance training on the composition of AChR subunits and their functional role in neurotransmission.

Agrin is a known key ligand that activates MuSK signalling via LRP4 binding. Homozygous *Agrn* mutant mice die in utero or are stillborn due to a lack of functional NMJs (Gautam et al., 1996), whereas suppression of LRP4 attenuates agrin‐induced MuSK activation and AChR clustering (Zhang et al., 2008). Furthermore, mice with genetic disruption of MuSK lack AChR clusters and show postnatal lethality (DeChiara et al., 1996), and postnatal MuSK conditional knockout mice lack NMJ maintenance (Hesser et al., 2006). Thus, MuSK signalling, including agrin, LRP4 and MuSK, plays crucial roles in maintaining NMJ integrity. To our knowledge, this is the first study to investigate the changes in MuSK signalling over time in response to muscle contraction that mimics acute resistance exercise and to demonstrate that muscle contraction dynamically alters the factors involved in MuSK signalling. Muscle contraction increased protein expression of MuSK and agrin, which are ligands of MuSK signalling. Additionally, muscle contraction increases *Rapsn* mRNA expression, which plays a key role in AChR clustering (Xing et al., 2019). However, muscle contraction transiently decreased the mRNA expression of the agrin receptor LRP4 and did not affect the expression of phosphorylated MuSK, an indicator of MuSK activity. This suggests that muscle contraction did not activate the MuSK signalling cascade but may have stimulated each factor independently. However, further studies are required to clarify the functional roles of changes in each factor.

### 4.2 Relationship between the changes in NMJ‐related factors and mTORC1 signalling activation

In the early recovery phase, mTORC1 activity increased at 24 h after muscle contraction, corroborating the results of a previous study (Ogasawara et al., 2016). Regarding NMJ‐related factors, *Agrn* mRNA expression increased at 3 and 6 h after muscle contraction, when mTORC1 activity peaked. A previous study demonstrated that the mTOR inhibitor CZ415 downregulated *Agrn* mRNA expression in fibroblasts (Woodcock et al., 2019), suggesting that mTOR signalling may be involved in *Agrn* expression. Since agrin is not only secreted by neurons, but also expressed in muscle cells (Lieth et al., 1992), activation of mTOR signalling may contribute to *Agrn* expression in myofibres. However, agrin protein expression did not increase during the acute recovery phase from muscle contraction in the present study. Therefore, considering that agrin protein expression increased during the late recovery phase from muscle contraction, when mTOR activity returned to the basal state, factors other than mTOR signalling may play a central role in the translational regulation of agrin. Alternatively, factors other than myofibres, such as motor neurons, may be a source of increased agrin content in the muscle tissue during late recovery from muscle contraction.

In contrast, AChR subunit gene expression increased in the late recovery phase from muscle contraction, when mTORC1 activity returned to the basal level. Although a recent previous study demonstrated that mTORC1 is involved in neuregulin‐1‐induced upregulation of protein expression of AChR subunits (Qiao et al., 2022), the discrepancy between the timing of mTORC1 activation and changes in AChR subunit gene expression suggest that mTORC1 activation in response to muscle contraction may have less of an effect on the changes in AChR gene expression after muscle contraction (Figure 9). Therefore, contraction‐induced mTORC1 activation was inhibited by rapamycin administration to clarify the contribution of muscle contraction‐induced mTORC1 activation on AChR subunit gene expression in vivo. The results showed that rapamycin decreased p70S6K phosphorylation indicating that administration of rapamycin inhibited mTORC1 activity, consistent with a previous study (Ogawara et al., 2016). However, rapamycin did not disturb the increase in AChR gene expression after muscle contraction. This suggests that muscle contraction increases AChR gene expression independent of mTORC1 activation, and other signalling pathways activated by muscle contraction contribute to the upregulation of AChR genes expression after muscle contraction. We also confirmed the mechanical relationship between mTORC1 activity and AChR gene expression in vitro and found that insulin‐induced activation of mTORC1 did not increase AChR gene expression. Instead, rapamycin increased *Chrnb*1 expression in vivo, and *Chrna*1, *Chrnd* and *Chrne* in vitro suggesting that mTORC1 may negatively regulate expression of AChR genes. This is consistent with a previous study which reported that rapamycin increased *Chrna*1 gene expression in myocytes (Altiok et al., 1997). Therefore, our current observation suggests that muscle contraction‐induced expression of AChR genes independently occurs with rapamycin‐sensitive mTORC1 activity.

The serine/threonine kinase Akt is activated by its phosphorylation at Thr308 and Ser473 (Manning & Toker., 2017). Mechanistically, Akt activates mTORC1 by repressing the TSC complex via the phosphorylation of tuberous sclerosis complex 2 (TSC2) (Saxton & Sabatini., 2017). Additionally, although Akt activation does not contribute to muscle contraction‐induced mTORC1 activation (Maruyama et al., 2020), it regulates HDAC4 expression, which is involved in synaptic gene expression after denervation (Yampolsky et al., 2010). A previous study demonstrated that inhibition of Akt activity induced by sustained activation of mTORC1‐mediated feedback in TSC knockdown mice impaired the nuclear translocation of HDAC4 and resulted in the lack of AChR turnover after denervation (Castets et al., 2019), suggesting that the Akt–HDAC4 signalling pathway plays a critical role in the AChR clustering. Consistent with a previous study, our study revealed that Akt phosphorylation levels increased immediately after muscle contraction (Ogasawara et al., 2016). Additionally, muscle contraction influenced the expression of HDAC4 in the whole lysate and nuclear fraction. These results suggest that muscle contraction activates the Akt–HDAC4 signalling pathway in the early recovery phase. However, the gene expression of AChR subunits did not change in the early recovery phase from muscle contraction. Thus, the role of the Akt–HDAC4 signalling pathway is limited to changes in NMJ‐related factors in response to muscle contraction.

However, a limitation of this study is that we used whole muscle, including both synaptic and extra‐synaptic regions, as the muscle sample; thus, we could not determine whether the changes in mRNA and protein expression occurred in the synaptic or extra‐synaptic regions. Further histochemical studies may provide answers to this question.

In conclusion, muscle contraction that mimics acute resistance exercise dynamically changed the gene expression of AChR subunits and factors involved in the MuSK signalling pathway. Furthermore, our study showed that mTORC1 and Akt‐HDAC4 activation in response to muscle contraction may also have less of an effect on changes in NMJ‐related factors. Particularly, we confirmed that an increase in AChR subunit gene expression after muscle contraction is independent of rapamycin‐sensitive mTORC1 activation. The results of this study support those of a previous study that demonstrated that resistance exercise changes AChR expression (Soendenbroe et al., 2020, 2022) and provides new insight into the molecular response to resistance exercise.

## AUTHOR CONTRIBUTIONS

Yuhei Makanae, Satoru Ato, Karina Kouzaki, Yuki Tamura and Koichi Nakazato designed the study conception. Yuhei Makanae, Satoru Ato, Karina Kouzaki and Yuki Tamura performed experiments. Yuhei Makanae and Satoru Ato analysed data. Yuhei Makanae, Satoru Ato, Yuki Tamura and Koichi Nakazato interpreted the results of the experiments. Yuhei Makanae and Satoru Ato prepared figures. Yuhei Makanae, Satoru Ato, Karina Kouzaki and Koichi Nakazato drafted the manuscript. All authors have read and approved the final version of this manuscript and agree to be accountable for all aspects of the work in ensuring that questions related to the accuracy or integrity of any part of the work are appropriately investigated and resolved. All persons designated as authors qualify for authorship, all those who qualify for authorship are listed.

## CONFLICT OF INTEREST

The authors declare no conflicts of interest.

## Data Availability

The data that support the findings of this study are available from the corresponding author upon reasonable request.
